# Exploring the Unmet Needs of Young Adults with Stroke in Australia: Can Technology Help Meet Their Needs? A Qualitative Study

**DOI:** 10.3390/ijerph20156450

**Published:** 2023-07-26

**Authors:** Dinah Amoah, Sarah Prior, Carey Mather, Matthew Schmidt, Marie-Louise Bird

**Affiliations:** 1 School of Health Sciences, University of Tasmania, Launceston, TAS 7250, Australia; 2 Tasmanian School of Medicine, University of Tasmania, Burnie, TAS 7320, Australia; 3 Australian Institute of Health Service Management, University of Tasmania, Hobart, TAS 7001, Australia

**Keywords:** post-stroke support, digital rehabilitation, young stroke, age specific resources, phenomenology, quality of life, Australia

## Abstract

The post-stroke needs of young adults with stroke are not being met, as most resources are tailored to older people with stroke. This includes technology-based applications, which are being used more frequently in stroke rehabilitation. There is limited data on technology usage to support the unique needs of young adults with stroke in Australia. This study aimed to explore the unmet needs of young adults aged 18–30 years with stroke and ascertain how technology can help meet those needs to improve quality of life and participation. Sixteen in-depth semi-structured interviews were conducted with young adults with stroke (*n* = 10), healthcare professionals (*n* = 3) and caregivers of young adults with stroke (*n* = 3). The interviews were transcribed verbatim and analyzed inductively. Five themes were generated: ‘Support for recovery’, ‘Availability of specific resources’, ‘Continuity of care’, ‘Adjustment’ and ‘Knowledge’. This study revealed the unique needs of young adults under 30 years with stroke who requested more targeted post-stroke support, age-specific resources and improved awareness on young stroke, with technology playing a pivotal role in all these interventions. We suggest co-designing technology-based solutions with young people after stroke to maximize their effectiveness in improving quality of life and participation in this unique cohort.

## 1. Introduction

Globally, the incidence of young stroke, defined as a stroke occurring among people younger than 70 years, is reported to have increased by 15% from 1990–2019 [1]. Similarly, there has been an increase in the incidence of stroke among young adults in Australia [2], with an estimated 24% of strokes occurring among young people <54 years old in 2020 [3]. Acute stroke in young adults (≤55 years) differs from older people (>55 years) in terms of risk factors, clinical features and outcomes [4]. Young stroke has been defined heterogeneously across many studies depending on the scope of the research, with common ranges from 18–55 years [5,6,7], 18–45 years [8], 24–62 years [9], 18–64 years [10], <65 years [11] and <50 years [12]. While there is no consensus on the definition of young stroke, current studies suggest that the upper limit is 65 years, consistent with the definition used in the global burden of stroke analyses [13].

The impact of stroke on the younger population can be profound. There is a risk of becoming disabled during the most productive stages of life and the potential for having to live with the sequalae of stroke longer than older people with stroke. Age-related life tasks, such as employment, schooling, relationships, parenting and active lifestyles, make young people with stroke face significantly more challenges across various areas of life than older people with stroke [6]. For instance, stroke often affects young people’s activities of daily living, such as a decline in social participation [7,14,15,16], driving [17] leisure activities [14,18], body image [19], sexual life [18,20], family relationships [18] and an inability to return to work [16,21,22]. In addition, a prospective national registry in Australia revealed that 74% of young adults aged 18 to 64 years with stroke are likely to have a lower health-related quality of life (HRQoL) compared to the older people with stroke [23].

The general impact of stroke may be more substantial in the younger (<30 years), sociologically unique cohort of adults with stroke when compared to those who are >30 years old in terms of quality of life. For example, an Australian study reported that people under 35 had a greater reduction in quality of life (more than 11 lost quality-of-life years) compared to those over 35 years (who had less than 11 lost quality-of-life years) [24]. Reduced quality of life (QoL) among young people is related to unemployment, fatigue, being single, depression and being functionally dependent [25,26]. These findings suggest that the quality of life of young people with stroke could be improved if the specific predictors to rehabilitation are identified early.

Young adults with stroke may have unique needs across a range of activities or spheres of life following their stroke [6]. Current resources available for people with stroke are generally tailored to older populations, with limited resources available to specifically support young people and their families after stroke [27]. Previous studies have reported a wide range of unmet needs for young adults with stroke, including lack of information [16,20,28,29], a lack of peer support services [29] and poor support with returning to work [18,20,28,30]. A recent international study specifically highlighted differences in the needs of young adults less than 35 years and above 35 years old with stroke [6]. This study revealed that young adults <35 years old with stroke did not want to seek help from healthcare professionals to address their needs, compared to those older than 35 years who had survived a stroke. Increased long-term unmet health needs in people with stroke are associated with a lower health-related quality of life [31,32]; meanwhile, most of the needs of these young adults with stroke are not met [6]. This finding suggests that the current resources available do not account for the variations in unmet needs of this cohort of young adults with stroke. Treating all patients equally, despite the fact their needs vary, creates an opportunity to identify the unique needs of young adults with stroke in order to meet age-specific requirements.

There have been several advances in the use of technology for stroke rehabilitation or general post stroke support over the last decade. Some areas where technology has been beneficial include serving as an alert for deviation from the normal [33], assisting survivors to engage in physical activity [34,35], increasing social participation, reducing boredom [36] and improving adherence to medications [36,37]. A range of technology-driven devices, such as video consultations, robotic-assisted devices, sensors, wearables and mobile apps have been employed to meet the specific needs of people with stroke. Although evidence suggests that young people are actively involved with technology, such as smartphones, for everyday life [38], limited studies have focused on how young adults with stroke utilize this technology. Of the few studies on young stroke, none have been performed specifically on the younger cohort of adults with stroke (<30 years). For instance, a recent survey on the attitudes of people with stroke towards the use of technology revealed that 100% of young adults (<65 years) with stroke expressed a preference for incorporating technology as a tool to improve their health, assist with self-management and rehabilitation exercises, and provide a source of information [39], thereby strengthening the necessity for developing technology-based solutions to address their unmet post-stroke needs.

Providing quality care to young adults with stroke requires dedicated and age-specific services and resources [40]. Although the current literature demonstrates the needs of young adults with stroke across a range of age groups, there is limited evidence to specifically highlight how to best meet the needs of those in the younger cohort. Therefore, this study aimed to explore the unmet needs of young adults with stroke aged 18–30 years old in Australia and understand how to best use technology to support their post-stroke needs.

## 2. Materials and Methods

This study is part of a larger study on supporting young stroke survivors through engaging messaging (SYSSTEM). The methods are reported in line with the consolidated criteria for reporting qualitative research (COREQ) guidelines [41]. The epistemological philosophical underpinning for the study was the constructivist–interpretivist paradigm. This paradigm was selected due to its ability to provide an in-depth understanding of the meanings that young adults with stroke attach to quality of life.

This study employed a phenomenological qualitative approach to explore and understand the unmet needs of young adults with stroke and the best way technology can help meet those needs. According to Crotty [42], the phenomenological approach focuses mainly on the phenomenon in a bid to understand the “object” of experience of it. Therefore, a phenomenological approach was appropriate to understand the experiences and perceptions of young adults with stroke, their caregivers and healthcare professionals with experience working with young adults with stroke.

### 2.1. Participant Selection

Participants including young adults with stroke, caregivers of young adults with stroke and healthcare professionals were recruited purposively to participate in the study. Eligible participants were selected based on the inclusion and exclusion criteria given in Table 1.

### 2.2. Recruitment

Recruitment for young adults with stroke in Australia occurred through advertisements by the Stroke Foundation of Australia on behalf of the research team. The same advertisement was utilized in social media through young adults with stroke groups in Australia on Facebook, Twitter and Instagram. Recruitment of caregivers occurred directly through the young adults with stroke. Recruitment flyers for the study were shared via email with professional network organizations including the Australian Association of Psychologists, Neurological Rehabilitation Group, Mental Health Professionals’ Network, Acute Stroke Nurses Education Network, and Speech Pathology Australia for the recruitment of healthcare professionals with experience working with young adults with stroke.

As part of the recruitment process, any young stroke participants who consented to be involved in the study underwent a screening assessment using the remote audio–visual version 8.1 of the Montreal Cognitive Assessment (MoCA) test [43] administered by a PhD candidate (DA) certified in the use of the screening tool. The MoCA screening tool was used as part of the recruitment process to help identify any potential participants unable to participate based on cognitive ability. The screening was administered via an online communication tool at a date and time arranged with the potential participants. This test consists of items that score up to 30 points, which took approximately 10 min to complete per participant. The MoCA was performed in seven steps, with the basics of this test including short-term memory, executable performance, attention, focus and more. While a score of ≥26 is classified normal, for our study, we included people with mild or moderate cognitive impairment; therefore, we used a cut-off score of 16.6 [44]. Potential participants with mild or moderate impairment, including those without cognitive impairment, were recruited.

This study was approved by the Tasmanian Human Research Ethics Committee, reference number H0026242.

**Figure 1:**
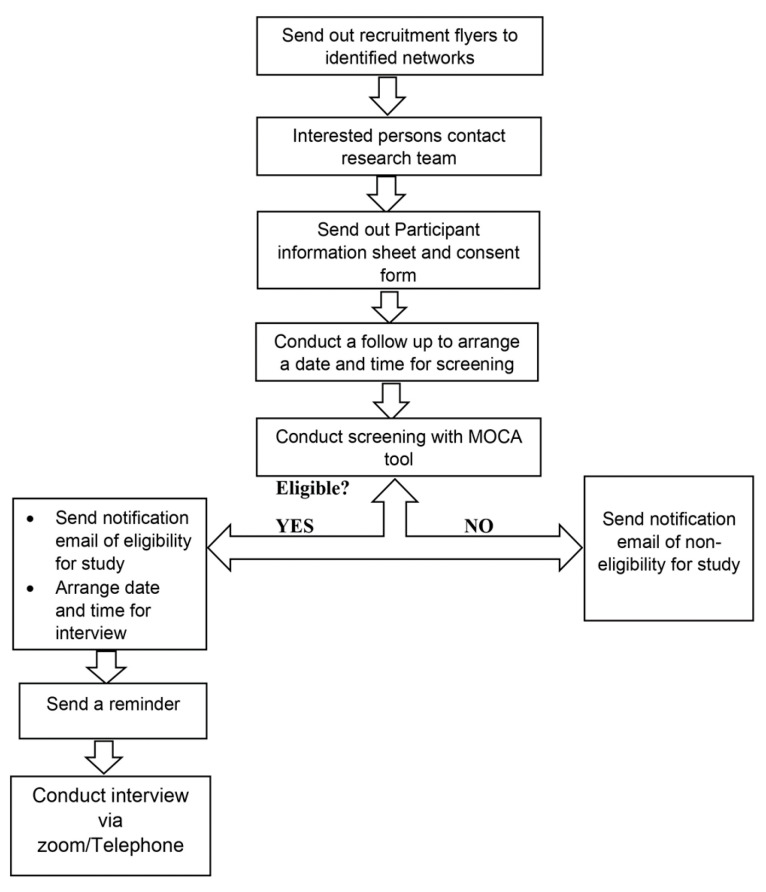
Recruitment flow chart.

The recruitment flow chart is presented in Figure 1.

### 2.3. Development of Interview Instrument

A semi-structured interview guide was co-designed based on literature, input, and feedback from the project’s advisory group comprising young adults with stroke and healthcare professionals with experience working with young adults with stroke. A pilot interview was conducted with a young adult with stroke who met the study’s criteria to ensure the rigor of the interview guide, as suggested by Padgett [45]. This interview was included in the study, as there were no changes made to the interview guide. The interview guide is provided in the Supplementary Material File S1.

### 2.4. Data Collection

Demographic information, including age, gender, marital status, occupation, location and length of time since stroke, was collected. The participants were then asked a series of semi-structured questions to identify and explore issues or problems they experienced since having a stroke. All interviews were conducted by a female PhD candidate (DA) who is a registered nurse with 12 years of experience in both clinical and public health specialties. DA did not have any prior knowledge of the participants or a relationship with the interviewees.

The interviews were conducted between September and November 2022 via an online provider or via telephone, depending on the participants’ preferences. Each interview commenced with an introduction to the study and information regarding privacy and consent. The interviews lasted for an average of 35 min (30–90 min), and each interview was audio recorded to ensure the information captured during the discussions was accurate. Prompts and questions were used to explore broad domains of the unmet needs of the participants. After the interview, a summary recap of the discussions was read to the participants to comment and make corrections where necessary. Data collection and analysis occurred simultaneously and progressed in phases until there were no new emerging themes (data saturation) [46].

### 2.5. Data Analysis

Data from the interviews were transcribed (DA) verbatim, then analyzed using NVivo software (version 1.6.1) [47]. A reflexive thematic analysis approach by Braun and Clarke [48] was adopted to code, generate themes, review themes and analyze data from the interviews inductively. A reflexive thematic analysis approach employs a rigorous, systematic approach to code and develop themes in a fluid and recursive manner with the inclusion of deep reflection on data other than a rigid approach [48]. A collaborative reflexive approach was employed with two researchers during the process of data coding and analysis in a bid to develop a richer interpretation of meaning, rather than seeking a consensus on meaning [48].

All the interview data were analyzed collectively (young adults with stroke, healthcare professionals and caregivers). This approach was utilized to ensure that the themes generated represented an in-depth and rich range of perspectives on the unmet needs of young adults with stroke. Employing thematic analysis, the researchers followed six procedures involving familiarizing themselves with data, generating firsthand codes, probing for themes, revising themes, defining and naming themes and reporting themes.

**Figure 2:**
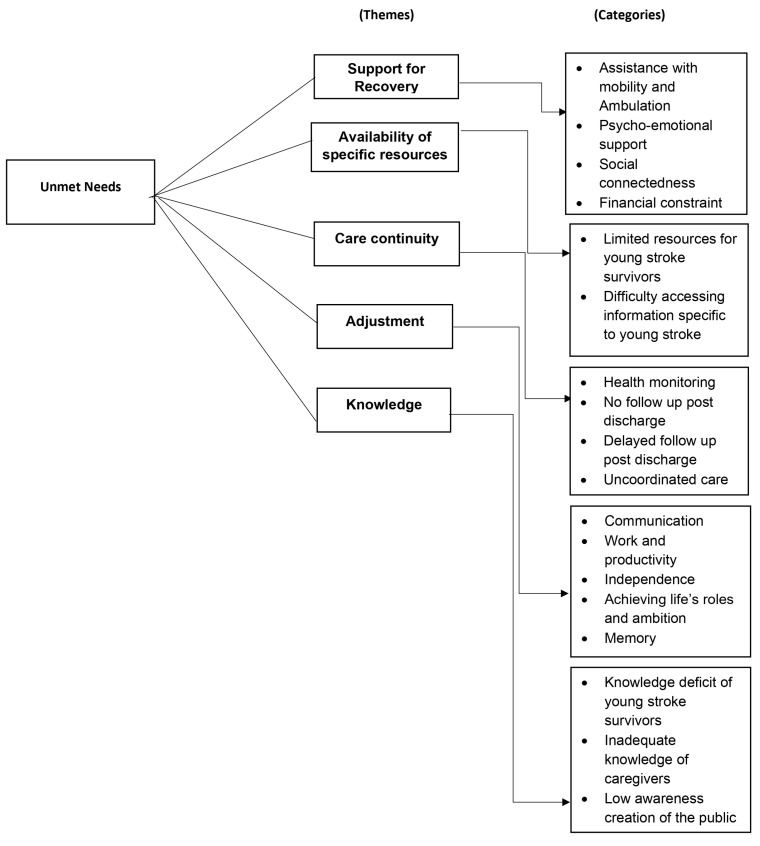
Thematic map of the unmet needs of young adults with stroke.

For the first phase, each interview transcript was read and re-read several times by DA and SP while listening to audio recordings to familiarize themselves with the data. During this phase, notes on interesting concepts were documented in a notebook. Secondly, after gaining an understanding of the interview transcripts, semantic and latent codes about the participants’ experiences were made. A mix of descriptive and in vivo first cycle coding methods were employed [49]. Thirdly, these initial codes were translated into themes. The themes captured information pertinent to the research question with shared patterned meaning within the data set [50]. Continuous reviewing of the coded data was done to identify areas of similarity and overlap between the codes. Further collapsing or clustering of similar codes was done to generate meaningful patterns in the data. Additionally, in this phase, relationships between the themes were considered to determine how to tell the story about the data. A thematic map was developed, which is presented in Figure 2.

During the fourth phase, the identified themes were reflected upon to ascertain connections between them and the entire dataset. In situations where the themes did not have a relationship with data, some codes were discarded, and others were amalgamated under a suitable theme. This process was done repeatedly until the themes captured the most relevant aspects of the data in relation to the research question. In the final phase, the major themes across all the 16 transcripts were reported narratively and supported by exemplar quotations from the participants.

## 3. Results

A total of 25 participants expressed interest in the study. However, four were >30 years old, one was from overseas and four did not turn up for the interview, either due to busy schedule or a change of decision to participate after submitting a signed consent form. Seven (44%) participants were recruited via Facebook and other social media platforms, six (38%) were recruited through a referral from a friend or someone else, two (13%) were recruited via the project’s advisory group and one (6%) was recruited via a professional association. Sixteen participants engaged in this study. They included young adults with stroke (*n* = 10), healthcare professionals (*n* = 3) and care givers (*n* = 3). The demographic data for each group is displayed in Table 2, Table 3 and Table 4.

The analysis of the interview data resulted in five main themes related to the unmet needs of young stroke survivors. These were: “support for recovery”, “availability of specific resources”, “care continuity”, “adjustment” and “knowledge”. A detailed description of each theme is given below.

### 3.1. Theme 1: Support for Recovery

The first theme, “support for recovery”, reflected discussions about what post-stroke support is most needed to improve or maintain quality of life for young adults with stroke. Most of the participants reported the presence of psycho–emotional problems in young adults with stroke, which were expressed in the form of anxiety, feelings of isolation, panic attacks, emotional outbursts, fear of recurrence of stroke and depression post-stroke. However, the participants felt that there was no or limited support from healthcare professionals about mental health, including psycho–emotional factors. Some of the young stroke participants utilized friends and/or family members to help them when they felt unable to cope with these feelings. Overall, the participants generally reported that they felt mental health was not appropriately included in support services. Healthcare providers also noted the importance of family support as part of a holistic model of care. They also suggested taking time to get to know young adults with stroke, their families and caregivers was an important step in understanding how best to support them during their recovery. Examples of young stroke participant statements in this theme are as follows:

“*I was never encouraged to go see anybody to work on the mental health side of things, feel like they put more of an emphasis on my physical well-being, which probably wasn’t tailored to how young people deal with strokes*.” (YSS 1)

“*I don’t think rehab should just be about getting someone’s body to be as healthy. I think the emotional side needs to be a little bit more part of it… I think lessening isolation is the quickest way to get quality of life*.” (YSS6)

“*As a young stroke survivor, you need to surround yourself with family and friends*.” (YSS5)

Another participant exemplar from a healthcare professional includes the following:

“*I think the best way you gather that information is really through conversations and spending time together really listening to what their life was like before their stroke, what it’s like now and kind of their goals and what they want their life to look like in the future*.” (HCP 2)

Support was a broad term used within discussions to refer to a range of needs. Financial assistance with specialist services was reported by some participants to be a limitation of support. Developing interpersonal relationships and re-learning to interact with people was flagged as being an important component of recovery that was not always included in rehabilitation or recovery services:

“*I think generally, the rehab system works really well to help people to basically function… to walk, get dressed to get out of bed, that kind of thing. But some of the high-level stuff like work and things like managing finances or relationships and recreation, I think there is less focus*.” (HCP3)

“*I couldn’t afford the services of a speech pathologist*.” (YSS 12)

Despite encountering difficulties, the young adults with stroke expressed optimism regarding their recovery and mentioned a variety of aspirations and goals to enhance their recovery. These objectives included building a family, excelling in academic and career pursuits and taking necessary precautions to prevent any future recurrences of stroke:

“*I would like to not have another stroke. Any, direction of not having another stroke would be great…. So my goal would be just to be better, have more energy and Just work towards bettering myself, I guess*.” (YSS 2)

“*Seeing my friends outside of Uni and just not be stroke survivor. But like, obviously, I am a survivor. I’ll always be a stroke survivor, but at the same time, like, I want to be more than that*.” (YSS 6)

When asked about the best ways to support young adults with stroke using technology, the participants discussed resources such as a tool for psycho–emotional support, a tool for learning and re-learning to use social media, video programs and interactivity in rehabilitation. One young adult with stroke stated:

“*Maybe in the form of an App so that young stroke survivor just type his feelings or emotions at any point in time and then someone will respond and give direction as to what to do or something like that*.” (YSS 8)

A caregiver participant also highlighted there may not be a place for technology in broadly supporting the needs of young adults with stroke, but that technology may be used to support specific types of disability, such as blindness or aphasia (text-to-voice applications).

“*I don’t see any tangible improvement by technology at the moment, not from what I’ve seen. There’s very certain scenarios that, you know, may help*.” (CS1)

### 3.2. Theme 2: Availability of Specific Resources

The availability of specific resources for young adults with stroke was seen as an important gap in current resources by the majority of the participants. The discussions focused on existing systems not meeting the needs of young adults with stroke. Some examples included the information available, support networks, medications prescribed and the procedures that were recommended or undertaken:

“*And I think that the infrastructure and the systems that are in place at the moment are very much geared towards older people that aren’t working ……You know, like, even coming down to what doctors do, like I was put on, like cholesterol medication, because that’s what they do when you have a stroke, because like older people have more cholesterol. But like, I didn’t have high cholesterol, but I guess it was part of the procedure so when you have a stroke, you get put on aspirin and all of this other medication and stuff. But like, I didn’t need that. A lot of the resources that the hospital gave me were all like, around older people*.” (YSS2)

In addition, most of the participants highlighted how stroke in young people is very isolating and becomes worse if not belonging to any social support group. The majority of the young adults with stroke were unaware of the existence of young stroke support groups available in Australia. Three of the young adults with stroke reported utilizing other social networks for young people with various health conditions as a way of reducing boredom and isolation. Even though young adults with stroke felt better belonging to young people’s social networks, a social support group solely for young adults with stroke was mentioned as the best alternative.

“*There’s this Stroke Association which I used to belong to, but I’m, sort of demotivated because most of the people in that group are older than me …… social media type thing groups specifically for people of my age with acquired brain injury and things like that. To me, that would probably be the best*.” (YSS 4)

“*The stroke survivors support group I found was not for young people, I joined once, and I saw that people were around 40s so I felt I could not share my experience, so I prefer a support group for young people*.” (YSS 12)

Conversely, a caregiver for a young adult with stroke felt that a social support group would not be appropriate due to the nature of the disability. For young stroke participants who reported belonging to a young stroke support group as a means of social connectedness, their needs were mostly not met, as the majority of the members were above 30 years old. As a result, they felt isolated even when belonging to a young stroke group:

“*But you know, having a stroke is very isolating as well. Especially as a young person, because if you’ve never been with someone or dealt with someone that’s had a stroke, it’s very confronting*.” (CS 1)

Furthermore, the majority of the participants mentioned difficulty in accessing information relevant for young adults with stroke in terms of support groups and how to navigate institutions post-stroke. Some participants outlined that they had to continuously search the internet to find resources relevant to them. Having a list of contacts on where to find information specific to young adults with stroke was suggested:

“*One of my clients, he’s doing a diploma and when you talk to student resources, if they have anything to help someone with a brain injury or a stroke, they just don’t have anyone that’s a specialist that understands really what’s going on, they just have like a very general mentor like a guidance counselor*.” (CS 2)

“*I think the main thing for me would be just being able to find the resources that I need like for my boy with xx disease knowing that there was a neurosurgeon in Australia, that did A sort of surgery that would be able to help him.*” (HCP1)

When asked about the most effective ways that technology can support or address this unmet need, the discussion primarily revolved around identifying resources that are well-suited and tailored to meet the specific needs of young adults with stroke, such as peer support via online providers that would specifically connect young adults less than 30 years old with stroke together.

“*So maybe they can create a platform where survivors can join and they can be talking to people posting maybe motivational stuff because it helps*.” (YSS 11)

Considering the stress faced by family members of people with stroke, a participant suggested making caregivers members of the young stroke support group:

“*I think if you had a social network for stroke survivors, specific for young stroke survivors that would be great….. it should also be for carers*.” (YSS3)

In addition, the young adults with stroke discussed feeling isolated and suggested various ways that technology could assist in addressing this problem:

“*Especially on Facebook or wherever, having the ability to reach out or find groups of young stroke survivors is very important to keep you from isolation, especially if you’re in a rural area, guess area where you can’t just go out and get to somewhere where there are people close your age*.” (YSS 6)

### 3.3. Theme 3: Care Continuity

The third theme, care continuity, embodies what the participants expressed regarding the care that young adults with stroke received or were receiving and their concerns about the progression of their health status during the stroke journey at home. Most participants reported that young adults with stroke did not usually receive any follow-up after discharge, while some received it, but at a very late stage. Some of the young adults with stroke outlined that they were neglected and left on their own, which led to feelings of isolation. Additionally, the participants highlighted a need for follow-up for all young adults with stroke, with or without visible disability, due to the ongoing psycho–emotional problems they experienced:

“*I think… I felt very isolated. But I think because I didn’t require any kind of rehabilitation after I had my stroke. I found, like, I was almost forgotten about. And it was sort of up to me to either do more research with the little pack that they gave me with the information about strokes. But that was kind of it. And I didn’t really have any other follow up. So I feel like younger people that I guess are in my situation don’t have that kind of after care.*” (YSS 2)

Additionally, one participant reported how the transition of care from pediatrics to adult stroke was uncoordinated, and that a telestroke did not exist in the state, resulting in an overwhelming burden on their family anytime they traveled for appointments:

“*I’ve had three different neurologist who don’t know me and they don’t know xx and they don’t know our story. And so having that continuity of care is a really big problem to fairly substantial one*.” (CS 3)

The participants also expressed how technology could assist address the gap in the provision of care post-discharge, such as the use of teleconferencing, mobile apps or other website applications to keep track of recovery and continue therapies.

An example quote from a young adult with stroke in relation to the use of technology is as follows:

“*Tracking your recovery so that you can actually see or know you’re recovering. It’s such a slow process. You want something that gives you feedback each day on how you’re going.*” (YSS 3)

In addition, some exemplars from the caregivers and healthcare professionals included the following:

“*Just like puzzle games, I guess, you know, the therapists would be able to use different kinds of computers and games to help further their therapies*” (CS 1)

“*Like using certain games that may be similar to the client’s ability to train and retrain certain movements*.” (HCP3)

### 3.4. Theme 4: Adjustment

The fourth theme, adjustment, represents the participants’ experiences that connoted problems related to their social and life’s roles such as returning to work, school, relationships, intimacy, recreation and dreams. Many of the participants either lost employment due the onset of stroke or lost a promotion, which consequently affected their financial capacity to provide for their needs. Due to fatigue, some participants had to change employment to accommodate their physiological or emotional challenges. This change was frustrating for some participants, as they might not have had any interest in their current job, but had no alternative. Therefore, it was difficult for some of the young adults with stroke to pursue the career they loved best due to the disability.

“*I lost the coordination of my work. I had to leave work for the first month…During that time, I was paid half of my salary*.” (YSS 10)

“*Like I didn’t work for like two months and that affected me financially*.” (YSS 11)

Some of the young adults with stroke had to defer their academic work or stop entirely. The participants reported that their future dreams were shattered due to stroke, such as dreams to engage in sports or particular professions.

“*I used to play footy when I was younger, but the doctors told me I shouldn’t play that anymore…. I was off work for about five or six weeks*.” (YSS 1)

“*So before my first stroke, I was always gonna go to uni and. I kind of got rid of that dream*.” (YSS 4)

Additionally, some of the parents found it difficult to adjust to the current situation of their child. The young adults with stroke wanted some form of independence in order to achieve some self-worth. Issues with maintaining and establishing relationships were reported by caregivers and healthcare professionals as a concern for young adults with stroke; however, no young adults with stroke saw this as a problem when they were asked.

“*I think a lot of young stroke survivors do struggle with relationships*.” (CS 1)

“*I’m 21 and can’t really have my parents’ advice on everything. Sometimes I just got to do things for myself*.” (YSS 6)

While it was difficult for the young adults with stroke to adjust to these life roles, some employers and institutions were cognizant of the disability and provided assistance to make life easier without putting stress on the young adults with stroke. Nevertheless, some of the young stroke participants reported not receiving any form of support after losing their job. Family, friends and engagement in social networks were reported to have facilitated their recovery, although some of the participants still struggled with communicating with friends and gaining independence in everyday activities of living:

“*My workplace supporting me by giving me what I needed for my job, you know, like, allowing me to reduce my hours or allowing me to, call in sick on the day without giving them a certificate*.” (YSS 2)

“*So I think one of the big challenges is that adjustment in life roles and being able to get back to normal life as much as possible*.” (HCP 2)

The participants reported various ways that technology could help support young adults with stroke to adjust to these life roles. These included tools to assist with self-management tasks and reminders for appointments/medications to assist with communication and organize their time to return to work or school. Some young adults with stroke stated:

“*It’s like remembering appointments and such and taking medications in the morning and things like that*.” (YSS 3)

“*So if there is an App that will help me deal with that, then I wouldn’t need a speech therapist*.” (YSS 12)

A caregiver also proposed an app to help establish intimacy and relationships:

“*And just maybe the same way you have dating Apps. But you can try and develop more dating apps for people with stroke*.” (CS 1)

### 3.5. Theme 5: Knowledge

Knowledge of a condition or situation is an important theme that depicts how individuals respond to situations. This theme encompasses the participants’ experiences regarding the knowledge level of young adults with stroke, healthcare professionals and the public on young stroke.

“*When I had my stroke, I didn’t know a lot about stroke*.” (YSS 11)

“*And I think most people aren’t aware that young people can have strokes. And then it’s actually quite common, you know*.” (YSS 2)

The participants reported how the lack of awareness of the public on the invisible signs of stroke creates unrealistic expectations of performance, which stresses young adults with stroke.

*It’s awkward because most people know that stroke happens to people who are in their late years, like from the 70s and beyond. But once you get stuck in your teens, your early ages, that is quite awkward. And people will begin looking at you in quite a different angle. …. so this awareness to everyone out there that Stroke can happen to anyone …*” (YSS 8)

The participants reported that inadequate knowledge of stroke in the young by the public led to stigmatization and worsened the recovery of young adults with stroke. One participant emphasized how the knowledge level of their partner on the general warning signs of stroke (F—face drooping, A—arm weakness, S—speech difficulty, T—time) helped with their recovery. Some young adults with stroke stated:

“*That was really hard for me because I went to a mainstream school and like, no one really understood me and most of them I guess treated me like how I used to be. So that was really like, hard and is a bit frustrating me*.” (YSS 4)

“*Like not many people are informed about stroke … So they should take a step and educate people about stroke so they become more informed*.” (YSS 11)

One healthcare professional highlighted the need for high-level topics to be discussed by healthcare professionals:

“*Some education of healthcare professionals can kind of broach some of these areas such as recreation, return to work, intimacy and sexuality because it doesn’t come up enough*.” (HCP3)

In addition, while the participants highlighted the knowledge gap on young stroke, some young adults with stroke had a fair idea about the prognosis of stroke among the young age groups:

“*Because you’re so young, your body also recovers a lot faster. So, someone that’s 70 years old, for example, may have a stroke and it may take them longer to recover from that*.” (YSS 1)

In a bid to ameliorate the issue of the knowledge gap coupled with its effects, the participants suggested a wide range of areas that technology could help in awareness creation strategies, and that this information should be up to date:

“*I think the clue to most chronic illnesses is providing up to date, information*.” (HCP 1)

“*I think raising awareness would be very important*.” (YSS 3)

## 4. Discussion

This study demonstrates a wide range of unmet needs of young (18–30 years) adults with stroke in Australia and highlights some useful ways that technology can help improve their quality of life and participation in areas such as psycho–emotional support, social connectedness, follow-up, adjustment to life’s roles and creation of awareness.

The findings indicated that young adults with stroke want a wide range of support for their recovery, with the need for psycho–emotional support being an important component. Depression is common among people with stroke as a result of social, cognitive and physical dysfunction decreasing the quality of life of affected persons [51]. While caregivers, friends and family played a role in supporting the ongoing mental health needs of some of the participants, other young adults with stroke resorted to their own personal strategies to keep them going. However, these approaches were inadequate, resulting in feelings of isolation. Consistent with this study, Bendz [52] suggested that while rehabilitation professionals focus on improving physical functioning, younger adults with stroke feel their concerns related to “loss of control, fatigue and fear of relapse” are not considered. Neglecting this is likely to negatively affect their full recovery and may affect the quality of life of young adults with stroke. It is essential to have structured psychological support services for young adults after stroke to enable them to deal effectively with any emotional and cognitive problems that may impact their quality of life and participation. This finding aligns with previous research conducted in various countries involving young adults with stroke aged between 18 and 67 years, which reported the necessity for psychological support services following stroke [7,27,53]. The participants proposed that leveraging technology-based resources, such as mobile applications or specialized websites tailored to address psychological and emotional challenges, would be highly beneficial in meeting this need.

The availability of specific resources tailored to young adults with stroke was another important area of unmet needs reported by the participants. This is consistent with the findings reported by Röding et al. [16], who described how the challenge of not having access to resources tailored to young adults after stroke leads to frustration, as current rehabilitation settings are not cognizant of the different needs of young stroke patients compared with older patients. Similarly, Medin et al. [30] found that younger adults with stroke perceived stroke services as mainly geared towards the needs of older rather than younger people due to a lack of available vocational support after stroke. Young adults with stroke had a preference for accessing age-relevant resources online as needed, rather than participating in physical groups like social support groups and healthcare services primarily attended by older young stroke survivors (>30 years).

This finding is consistent with an international young stroke study which reported different needs for people <35 years compared to those above 35 years in relation to preferences for seeing healthcare professionals [6]. Our study emphasizes the importance of having a separate social support group for younger cohorts (<30 years) of young adults with stroke through the use of social media, mobile apps or any technology-based platform. The participants also suggested the development of a list of contacts of important resources specific to young adults with stroke which could be shared with young adults with stroke through any technologically driven mode, either during rehabilitation or after discharge.

Generally, when provided well, continuity of care has been shown to reduce all causes of mortality [54], improve patients’ experience of care and reduce preventable hospitalization [55]. Despite the benefits of continuity of care, most of the respondents verbalised that young adults with stroke do not usually receive any follow-up visit. For the participants who did receive a follow-up, it was delayed for over a year. The absence or delay of follow-up left the participants feeling isolated and unaware of the outcomes of their condition, causing more anxiety and depression, with some thinking they were “forgotten about”. This finding confirms previous studies on young stroke (18–67 years), which have posited how young adults with stroke did not receive adequate therapy post-stroke or any follow-up [28,53]. To address this, the participants suggested having a structured and early follow-up system employing a wide range of technology-based approaches such as online consultation and telestroke for all young adults with stroke, including those without any visible disability. A previous randomised controlled trial reported improved psychosocial status and improved exercise capacity among type 2 diabetes mellitus patients who were engaged in telerehabilitation [56], supporting our findings. Furthermore, Pitt et al. [57] reported improved communication, participation and quality of life for people with chronic aphasia who engaged in telerehabilitation. Early follow-up for young adults with stroke is important to improve their recovery [7].

In a bid to address the problem of adjustment, the participants proposed the use of technology to assist with adjustment to social roles, such as recreation, psychosocial support services, management of work, schoolwork, self-management and financial assistance. This finding is consistent with a cross-sectional study among a working age (18–64 years) group of people with acquired brain injuries (ABI), which revealed a significant likelihood of return to work among ABI patients who utilized everyday technology such as computers, internet services and smartphones [58]. Similarly, incorporating a medication reminder function within an app has been identified as a potential technological solution to reduce the cognitive challenges faced by individuals with stroke [59]. The impact of stroke on career consequently affects one’s finances, resulting in an inability to provide for their needs. In addition, returning to school, being able to undertake ADLs independently and participating in social events such as sports were reported as areas of need. These problems consequently have a negative effect on the survivor and family if structured systems are not in place to assist with adjustment. Although the caregivers and healthcare professionals outlined intimacy and relationship problem as being an area of concern for young adults with stroke, it is surprising that the young adults with stroke interviewed did not mention any difficulties regarding their relationships. This finding contradicts another systematic review and thematic synthesis which revealed changes in relationships between people with stroke (>18 years) and their partners, requiring the person with stroke to adjust to living with a changed body [60]. Our study’s finding could indicate that the young adults with stroke did not want to talk about their issues with relationships, or that this perceived need of young adults with stroke may not match the actual need of young adults with stroke. A reduced quality of life in young survivors is associated with dependence, depression, being single, fatigue and being unemployed [25]. Similarly, an interview with 43 young (<60 years) adults with stroke revealed how important returning to work is to young adults with stroke. It is also a measurement for successful recovery [22].

Education is an important tool used to create awareness regarding health and disease conditions, including stroke, to achieve behavior changes [61]. The knowledge inadequacy reported in this study confirms evidence which reveals how a lack of knowledge regarding stroke contributes to unhealthy lifestyles and places patients at risk of stroke recurrence [62]. To address this knowledge gap, the participants suggested the implementation of educational programs in schools as a means of raising public awareness. This study therefore proposes a counselling session for caregivers to re-orientate them regarding alternate endeavors for young adults with stroke in the event of disability. While it is important to create awareness on stroke, the participants emphasized the significance of providing contemporary information. Regarding the content of education, the healthcare professionals suggested educating young adults with stroke on high-level topics such as sexuality, leisure and intimacy. The use of games, apps and websites were proposed as technology-based tools for delivering this education to increase awareness of young stroke. This is consistent with studies that indicate how the utilization of mobile applications has the capacity to enhance the understanding of risk factors among young people (≤55 years) with stroke and contribute to an improved quality of life [37,63].

Although technology was mentioned to have a place in addressing unmet needs, considering varying areas of disability with stroke, the participants emphasized the need for the tool to possess some specifications such as being cost effective, accessible to all levels of dysfunction, having options for different languages and being tested by people with aphasia. To optimize the efficacy of the use of any technology-based tool, a co-design with young adults with stroke was recommended. These findings are consistent with a previous study emphasizing the benefits of co-design approaches for tackling intricate issues and enhancing stakeholder involvement [64].

### 4.1. Strength of Study

Currently, there are no studies that have qualitatively explored the unmet needs of the younger cohorts (18–30 years) of young adults with stroke in Australia and how technology could support their recovery and well-being. In view of this, the strength of this study lies in its contribution to the body of literature on young stroke in Australia and internationally.

### 4.2. Limitations

This study cannot be generalized, as purposive sampling was used to select research participants, and the opinions and experiences of participants differ from one setting to another. However, the findings of this study contain detailed and contextual information. In addition, there is a likelihood of participation bias, as the participants were not recruited from stroke units, and a confirmation of a diagnosis of stroke was not undertaken. To address this limitation, similar studies should consider recruiting participants from rehabilitation centers or ascertain a way of confirming diagnosis through their medical records.

## 5. Conclusions

This study highlights the unique needs of young adults with stroke under 30 who requested more targeted post-stroke support, age-specific resources and improved awareness of young stroke, with technology playing a pivotal role in all these interventions. Developing technological solutions in collaboration with young people after stroke can maximize their relevance and effectiveness in improving quality of life and participation in this unique cohort.

## Figures and Tables

**Table 1:** Inclusion and exclusion criteria.

Inclusion Criteria	Exclusion Criteria
Young adults with stroke aged 18–30 years who had their stroke when they were less than 25 years old	Young adults with stroke less than 18 years old or over 30 years old
Self-reported diagnosis of stroke	Young adults with stroke who had a stroke when they were >25 years old
Caregivers of young adults with stroke (aged 18–30) who had their stroke when they were <25 years old	
Healthcare professionals such as nurses, neurologists, neuropsychologists, paediatricians, speech therapists, occupational therapists, general physicians, physiotherapists and social workers who have at least 12 months of experience working with young stroke survivors	Severe cognitive impairment
Can understand and respond to questions in English	Individuals who speak a first language other than English and are unable to understand and respond to questions in English. This is important because of the cost involved with hiring a translator and their inability to join focus groups with other English speakers. Additionally, sometimes translations may fail to truly represent the actual experience or views of the respondent due to language and cultural complexities.
Resident in any Australian states or territories	Resident in countries outside Australia

**Table 2:** Demographic characteristics of the young adults with stroke who were interviewed.

NO	Study ID	Current Age	Marital Status	Employment at Interview	Type of Stroke	Gender	Time since Stroke (Years)	Interview Mode	Location
1	Y.S.S 1	25	In a relationship	Project officer	Ischaemic	M	3	Telephone	Northern Territory
2	Y.S.S 2	26	Married	Administrator	Ischaemic	F	1	Telephone	Tasmania
3	Y.S.S 3	24	Single	Studying	Not specified	M	3	Zoom video	Tasmania
4	Y.S.S 4	26	Single	Unemployed	Not specified	M	15	Zoom video	Queensland
5	Y.S.S 5	21	Single	Studying	Haemorrhagic	M	3	Zoom video	Queensland
6	Y.S.S 6	21	In a relationship	Studying	Haemorrhagic	F	4	Zoom video	South Australia
7	Y.S.S 8	22	In a relationship	Freelancer	Not specified	M	4	Zoom video	Victoria
8	Y.S.S 10	27	In a relationship	Employed	Not specified	M	1	Zoom video	New South Wales
9	Y.S.S 11	26	Not specified	Employed	Not specified	F	2	Zoom video	Tasmania
10	Y.S.S 12	22	In a relationship	Employed	Not specified	F	1	Zoom video	Victoria

**Table 3:** Demographic characteristics of the interviewed caregivers.

No.	Study ID	Type of Support	Gender	Years of Experience	Interview Mode	Location
1	CS. 1	Disability support worker	M	1	Telephone	Queensland
2	CS. 2	Child	F	19	Telephone	Queensland
3	CS. 3	Disability support worker	M	8	Telephone	Tasmania

**Table 4:** Demographic characteristics of the interviewed healthcare professionals.

No.	Study ID	Role	Gender	Years of Experience	Interview Mode	Location
1	HCP. 1	Paediatrician	M	32	Telephone	New South Wales
2	HCP. 2	Speech and Language Pathologist	F	22	Zoom video	Tasmania
3	HCP. 3	Physiotherapist	F	5	Zoom video	Tasmania

## Data Availability

The data are contained within the article and Supplementary Materials.

## Supplementary Materials

The following are available online at https://www.mdpi.com/article/10.3390/ijerph20156450/s1, Supplementary Material File S1: Interview Guide Questions, Supplementary Material File S2: Consolidated criteria for Reporting Qualitative research (COREQ) checklist.
