# Supplementary material for: Exploring the Unmet Needs of Young Adults with Stroke in Australia: Can Technology Help Meet Their Needs? A Qualitative Study
Source: Int J Environ Res Public Health. 2023 Jul 26;20(15):6450. doi: 10.3390/ijerph20156450 (PMC10418600; doi:10.3390/ijerph20156450)
Supplement: Supplementary file 1 [file ijerph-20-06450-s001.zip › Supplementary File S1- Interview guide.pdf]

INTERVIEW GUIDE - YOUNG STROKE SURVIVORS

Introduction

Thank you for taking the time to participate in this study. My research, which is part of my doctoral studies, seeks to hear your story as a young person with stroke including whether you feel that technology may be a useful tool for you post-stroke. Before we begin, I need to inform you of the guidelines for this discussion.

Guidelines for 1:1 Interview

- The discussion will be centered around four main themes:
  - Unmet needs of young stroke survivors
  - Solutions to address unmet needs of young stroke survivors
  - Availability of technology for young stroke survivors
  - The potential role of technology to improve quality of life and participation in society for young stroke survivors
- You are being reminded that the discussions will be audio recorded so that it can be listened to again to ensure information captured during the discussions are accurate
- Information provided is confidential, will not be using real names or identifying information without permission
- There are no right, or wrong answers
- Please feel free to make notes about anything you'd like to comment on as our discussion gets going
- The discussion is expected to last 30-90 minutes
- Please feel free to discuss your experiences and perceptions as a young stroke survivor

PART I: DEMOGRAPHIC CHARACTERISTICS

Before we get into some specific questions, can you please tell me something about yourself?

[**Prompt**: You can mention your first name, age, marital status, occupation, state, type of stroke, how long you have had your stroke]

PART 2: UNMET NEEDS OF YOUNG STROKE SURVIVORS

I would like to have a general picture of your experiences being a young person with stroke.

1. How would you describe your life after stroke?
   - **Promp**t: what is it like to be a young person with stroke
   - **Prompt:** In the early days following your stroke vs when transitioning from hospital to home vs. when reconnecting with your community
   - **Prompt**: Do you have any problems; You can share problems with walking, moving your arm or leg, talking, swallowing, eating, feeling tired, moody, depressed, anxious, lacking confidence, losing your identity, or feeling isolated
2. How has your experience as a stroke survivor impacted your life?
   - **Prompt:** You can share its impact on participation, education, employment, relationship, family role, independence

PART 3: SOLUTIONS TO ADDRESS UNMET NEEDS OF YOUNG STROKE SURVIVORS

This session will explore how best you want your identified unmet needs to be met.

3. What are the three things that could have been improved or you want to be improved?
   - **Prompt:** In the early days following your stroke vs when transitioning from hospital to home vs. when reconnecting with your community
   - **Prompt**: independence, financial problems, isolation,
4. Do you have any suggestions for improving these needs?
   Prompt: financial support, technology-based solution, social network, provision of factsheets
5. What hopes/dreams do you have for the next 12months?
   - **Prompt**: How you perceive yourself in the stroke journey
   - **Prompt**: Are you heading where you want to be heading?
   - **Prompt**: What has helped you get on or off the right path
   - **Prompt**: what roadblocks have you experienced
6. What do you need to achieve those dreams/hopes?

PART 4: AVAILABILITY OF TECHNOLOGICAL RESOURCES FOR YOUNG STROKE SURVIVORS

We would like to ask you about any resources that you might be aware of for people who have had a stroke and whether these have been helpful for you.

7. Can you tell me about any technological resources you know that are available to assist young stroke survivors in their everyday life?
   - **Prompt**: mobile app, telehealth, game, videos.
8. Are you using any technology such as smartphones, tablets to assist you with your activities of daily living?
   - If yes, can you describe the type of technology you are using and what it is used for?
   - If no, what are the barriers to using technology in your everyday life

9. In general how would you describe the technological resources available for young stroke survivors?

- **Prompt:** You can talk about its accessibility to young stroke survivors
- **Prompt:** You can talk about its effectiveness to make the greatest impact in the life of young stroke survivors

9. PART 5: ROLE OF TECHNOLOGY TO IMPROVE QUALITY OF LIFE OF YOUNG STROKE SURVIVORS

In this last session, we would like to explore the role technology might play in improving the quality of life of young stroke survivors

10. Would you be interested in using technology such as phones, smart watches, tablets that will assist you with the performance of activities of daily living?
    - **Prompts:** what drives your interest
    - **Prompt**: How will it be helpful
    - **Prompt**: Do you think it will be useful to the general young stroke survivors
11. If we are developing something for use technologically, what type would help in making the greatest impact in your life?
    - **Prompt**: would you prefer smartphones, tablets,
    - **Prompt**: what informs your choice
    - **Prompt**: what would you like your technology to do for you? eg. Sending reminders, linking to social network

Closing Remarks

I hope you found the discussion interesting and worthwhile. Thank you for your participation and for sharing so openly.

INTERVIEW GUIDE - HEALTHCARE PROFESSIONALS

Introduction

Thank you for taking the time to participate in this study. My research, which is part of my doctoral studies, seeks to hear about your experiences working with young people with stroke, their unmet needs, and to find out how these needs can be met to improve their quality of life and participation. I need to inform you of the guidelines for this discussion before we begin.

Guidelines for 1:1 Interview

- The discussion will be centered around four main themes:
  - Unmet needs of young stroke survivors
  - Solutions to address unmet needs of young stroke survivors
  - Availability of Technology for young stroke survivors
  - The potential role of technology to improve quality of life and participation in society for young stroke survivors
- You are being reminded that the discussions will be audio recorded so that it can be listened to again to ensure information captured during the discussions are accurate
- Information provided is confidential, will not be using real names or identifying information without permission
- There are no right or wrong answers
- Please feel free to make notes about anything you'd like to comment on as our discussion gets going
- The discussion is expected to last 30-90 minutes
- Please feel free to discuss your experiences and perceptions of working with young stroke survivors

PART I: DEMOGRAPHIC CHARACTERISTICS

Before we get into some specific questions, can you please tell me something about yourself?

[**Prompt**: Please state your first name, location (state), your professional background (neurologist, nutritionist, etc.), how long you have worked with stroke patients]

PART 2: UNMET NEEDS OF YOUNG STROKE SURVIVORS

I would like to have a general picture of your experiences working with young persons with stroke.

12. How would you describe your experience working with young persons with stroke?
    - **Promp**t: what age range among the young people with stroke do you usually see
    - **Prompt:** what gender among the young stroke clients usually report for care and treatment
    - **Prompt:** what type of stroke is usually predominant among the younger group
    - **Prompt**: Do they report early or late to the healthcare facility
13. Can you share with me some problems or disabilities that young stroke clients usually present with?
    Prompt: You can share problems with walking, moving your arm or leg, talking, swallowing, eating, feeling tired, moody, depressed, anxious, lacking confidence, losing your identity, or feeling isolated
14. How do these problems affect your management and care for the stroke survivors in improving their quality of life?

- **Prompt:** You can share its impact on self-care, nutrition, medication management, development of secondary co-morbidities, independence

PART 3: SOLUTIONS TO ADDRESS UNMET NEEDS OF YOUNG STROKE SURVIVORS

This session would explore the needs of young stroke survivors and the best way these needs are met by health professionals

15. Can you share with me the top three unmet needs that the young stroke survivors report?
    - **Prompt**: independence, financial problems, isolation,
16. How are you able to know the unmet needs of young stroke survivors?
    - **Prompt**: Do you find out during admission
    - **Prompt**: Do you find out during follow up after admission
    - **Prompt**: Do you find out from their caregivers
17. How do you manage or deal with each of the problems reported?
    - **Prompt**: Do you focus on clinical interventions (medication management)
    - **Prompt:** Do you focus on other public health interventions such as referring to social support groups, counselling, assistance to return to work, return to school, independence
18. Can you share with me the outcome of such interventions in making their life better?
    - **Prompt**: Are they usually successful or not
    - **Prompt**: How do you know if your interventions are appreciated by the young stroke survivors or not

PART 4: AVAILABILITY OF TECHNOLOGICAL RESOURCES FOR YOUNG STROKE SURVIVORS

We would like to find out your knowledge of the already existing resources and how helpful they have been.

19. Can you tell me about any technological resources you know that are available to assist young stroke survivors in their everyday life?
    Prompt: mobile app, telehealth, game, videos
20. How would you describe the technology available for young stroke survivors?
    Prompt: You can talk about its accessibility (geographical, financial, usability) to young stroke survivors
    Prompt: you can talk about its effectiveness to make the greatest impact in the life young stroke survivors

PART 5: ROLE OF TECHNOLOGY TO IMPROVE QUALITY OF LIFE OF YOUNG STROKE SURVIVORS

In this last session, we would like to explore the role technology used for everyday life might play in improving the quality of life of young stroke survivors

21. Would you recommend the use of technology to make the greatest impact in the life of young stroke survivors?
    - **Prompts:** what drives your interest
    - **Prompt**: How will it be helpful
    - **Prompt**: Do you think it will be useful to the general young stroke survivors
22. If we are developing something for use technologically, what type do you think would help you in the effective management of young stroke survivors to improve their quality of life?
    - **Prompt**: Should it be smartphones, tablets,
    - **Prompt**: what informs your choice

- **Prompt**: what would you like the technology to do for young stroke survivors? eg. Sending reminders, linking to social network

Closing Remarks

I hope you found the discussion interesting and worthwhile. Thank you for your participation and for sharing so openly.

INTERVIEW GUIDE - CARERS FOR YOUNG STROKE SURVIVORS

Introduction

Thank you for taking the time to participate in this study. My research, which is part of my doctoral studies, seeks to hear about your experiences working with young people with stroke, their unmet needs, and to find out how these needs can be met to improve their quality of life and participation. I need to inform you of the guidelines for this discussion before we begin.

Guidelines for 1:1 Interview

- The discussion will be centered around four main themes:
  - Unmet needs of young stroke survivors
  - Solutions to address unmet needs of young stroke survivors
  - Availability of Technology for young stroke survivors
  - The potential role of technology to improve quality of life and participation in society for young stroke survivors
- You are being reminded that the discussions will be audio recorded so that it can be listened to again to ensure information captured during the discussions are accurate
- Information provided is confidential, will not be using real names or identifying information without permission
- There are no right or wrong answers
- Please feel free to make notes about anything you'd like to comment on as our discussion gets going
- The discussion is expected to last 30-90 minutes
- Please feel free to discuss your experiences and perceptions of working with young stroke survivors

PART I: DEMOGRAPHIC CHARACTERISTICS

Before we get into some specific questions, can you please tell me something about yourself?

[**Prompt**: You can mention your first name, age, occupation, relationship to the stroke survivor, how long you have supported young people with stroke]

PART 2: UNMET NEEDS OF YOUNG STROKE SURVIVORS

I would like to have a general picture of your experiences caring for a young person with stroke.

- How would you describe your experience caring for young persons with stroke?
  Prompt: what is it like to care for a young person with stroke
  Prompt: During which transitional period did you start caring for the young stroke survivor, is it in the early days following stroke or when transitioning from hospital to home or when reconnecting with the community
  Prompt: What problems do they have; You can share problems with walking, moving your arm or leg, talking, swallowing, eating, feeling tired, moody, depressed, anxious, lacking confidence, losing your identity, or feeling isolated
- How do these problems affect your care for the stroke survivors in making the greatest impact in their life?
- **Prompt:** You can share its impact on participation, self-care, nutrition, education, employment, relationship, family role, independence

PART 3: SOLUTIONS TO ADDRESS UNMET NEEDS OF YOUNG STROKE SURVIVORS

This session would explore the needs of young stroke survivors and the best way these needs are met.

- What are the three things that could have been improved or you want to be improved for young stroke survivors?
- **Prompt:** In the early days following stroke vs when transitioning from hospital to home vs. when reconnecting with your community
- **Prompt**: independence, financial problems, isolation,
- Do you have any suggestions for improving these needs?

Prompt: financial support, technology-based solution, social network, provision of factsheets

- What hopes/dreams do you have for the next 12months as a carer for young stroke survivors?
- **Prompt**: How you perceive the young stroke survivor in the stroke journey
- **Prompt**: Are they heading where you want them to be heading
- **Prompt**: Are you heading where you want to head with their care
- **Prompt**: What has helped you get on or off the right path to caring for young stroke survivors
- **Prompt**: What roadblocks have you experienced in caring for young stroke survivors
- What do you need to achieve those dreams/hopes?

PART 4: AVAILABILITY OF TECHNOLOGICAL RESOURCES FOR YOUNG STROKE SURVIVORS

We would like to ask you about any resources that you might be aware of for people who have had a stroke and whether these have been helpful.

- Can you tell me about any technological resources you know that are available to assist young stroke survivors in their everyday life?
- **Prompt**: mobile app, telehealth, game, videos.
- Are you using any technology such as smartphones, tablets to assist young stroke survivors with their activities of daily living?
- If yes, can you describe the type of technology you are using and what it is used for?
- If no, what are the barriers to using technology for their everyday life

9. In general how would you describe the technological resources available for young stroke survivors?

- **Prompt:** You can talk about its accessibility to young stroke survivors
- **Prompt:** You can talk about its effectiveness to make the greatest impact in the life of young stroke survivors
- **PART 5: ROLE OF TECHNOLOGY TO IMPROVE QUALITY OF LIFE OF YOUNG STROKE SURVIVORS**

In this last session, we would like to explore the role technology might play in improving the quality of life of young stroke survivors

- Would you recommend the use of technology such as smart phones, tablets, smart watches to make the greatest impact in the life of young stroke survivors?
  - **Prompts:** what drives your interest
  - **Prompt**: How will it be helpful
  - **Prompt**: Do you think it will be useful to the general young stroke survivors
- If we are developing something for use technologically, what type do you think would help you in the effective care of young stroke survivors to make the greatest impact in their life?
  - **Prompt**: would you prefer smartphones, tablets,
  - **Prompt**: what informs your choice

- **Prompt**: what would you like the technology to do for young stroke survivors eg. Sending reminders, linking to social network

Closing Remarks

I hope you found the discussion interesting and worthwhile. Thank you for your participation and for sharing so openly.
